# Health financing and integration of urban and rural residents’ basic medical insurance systems in China

**DOI:** 10.1186/s12939-017-0690-z

**Published:** 2017-11-07

**Authors:** Kun Zhu, Luying Zhang, Shasha Yuan, Xiaojuan Zhang, Zhiruo Zhang

**Affiliations:** 1 Chinese Academy of Medical Sciences and Peking Union Medical College Institute of Medical Information, No. 3 Yabao Road, Chaoyang District, 100020 Beijing, People’s Republic of China; 2 School of Public Health, Fudan University, No. 138 Yixueyuan Road, Xuhui District, 200032 Shanghai, People’s Republic of China; 3 Shanghai Jiao Tong University School of Public Health, No. 227 South Chongqing Road, Huangpu District, 200025 Shanghai, People’s Republic of China

**Keywords:** Health financing, Equity, Public finance, Medical insurance, Universal health coverage

## Abstract

**Background:**

China is in the process of integrating the new cooperative medical scheme (NCMS) and the urban residents’ basic medical insurance system (URBMI) into the urban and rural residents’ basic medical insurance system (URRBMI). However, how to integrate the financing policies of NCMS and URBMI has not been described in detail. This paper attempts to illustrate the differences between the financing mechanisms of NCMS and URBMI, to analyze financing inequity between urban and rural residents and to identify financing mechanisms for integrating urban and rural residents’ medical insurance systems.

**Methods:**

Financing data for NCMS and URBMI (from 2008 to 2015) was collected from the China health statistics yearbook, the China health and family planning statistics yearbook, the National Handbook of NCMS Information, the China human resources and social security statistics yearbook, and the China social security yearbook. “Ability to pay” was introduced to measure inequity in health financing. Individual contributions to NCMS and URBMI as a function of per capita disposable income was used to analyze equity in health financing between rural and urban residents.

**Results:**

URBMI had a financing mechanism that was similar to that used by NCMS in that public finance accounted for more than three quarters of the pooling funds. The scale of financing for NCMS was less than 5% of the per capita net income of rural residents and less than 2% of the per capita disposable income of urban residents for URBMI. Individual contributions to the NCMS and URBMI funds were less than 1% of their disposable and net incomes. Inequity in health financing between urban and rural residents in China was not improved as expected with the introduction of NCMS and URBMI. The role of the central government and local governments in financing NCMS and URBMI was oscillating in the past decade.

**Conclusions:**

The scale of financing for URRBMI is insufficient for the increasing demands for medical services from the insured. The pooling fund should be increased so that it can better adjust to China’s rapidly aging population and epidemiological transitions as well as protect the insured from poverty due to illness. Individual contributions to the URBMI and NCMS funds were small in terms of contributors’ incomes. The role of the central government and local governments in financing URRBMI was not clearly identified. Individual contributions to the URRBMI fund should be increased to ensure the sustainable development of URRBMI. Compulsory enrollment should be required so that URRBMI improves the social medical insurance system in China.

## Background

According to a report by the World Health Organization (WHO) in 2000, the performance of China’s health system was far from expectations. The ranking of equity in health financing was listed as fourth from last in the world [1]. One of the main causes of this phenomenon was that most of China’s residents were not covered by any social health security system and they paid for health care out of pocket. The second Chinese national health survey in 1998 confirmed that 44.8% of urban citizens and 79.1% of rural residents in China were not covered [2].

To improve equity in health financing and especially to protect rural Chinese residents from impoverishment by illness, in 2002, China’s government decided to establish a wide and shallow social medical insurance system called New Cooperative Medical Scheme (NCMS), which covered rural residents [3]. In 2003, the Ministry of Health (MOH), the Ministry of Finance (MOF), and the Ministry of Agriculture (MOA) jointly issued directives on the establishment of NCMS and determined that public finance, individual contributions and collective support would be the main financing sources [4]. NCMS began as a pilot in 2003 and rapidly expanded to rural areas in China. In 2007, nearly all rural counties and more than 85% of rural residents were covered by NCMS, which had voluntary enrollment [5]. Based on their experience with NCMS, China’s government began to establish a new medical insurance system called the Urban Residents’ Basic Medical Insurance (URBMI) system, which covered urban residents who were not covered by the urban employees’ basic medical insurance system. In 2007, the State Council of China issued directives on the pilots of the urban residents’ basic medical insurance system and determined that families would be the key contributors to its financing and that public finance would also subsidize it to some extent [6].

With several years of development, NCMS and URBMI operated smoothly and laid the foundation for China to approach universal health coverage. However, as population migration became increasingly common in China, some migrants were covered by both NCMS and URBMI, which created financial burdens on individuals and public finance [7].

To avoid overlap in NCMS and URBMI coverage; improve the equity, sustainability and efficiency of NCMS and URBMI; and advance the development of universal health coverage (UHC), the State Council of China issued the “Opinion on the integration of basic medical insurance systems between urban and rural residents” in January 2016 and required that NCMS and URBMI be integrated into the urban and rural residents’ basic medical insurance system (URRBMI) based on the principle of six unifications of coverage, financing policy, benefit packages, lists of drugs and services, contract suppliers and fund management [8].

Financing policy is one of the six key components of the integration process. However, the document did not describe in detail how to integrate the financing policies of NCMS and URBMI. The role of public finance in establishing URRBMI for central and local government, how to adjust the contributions of individuals and public finance to fund URRBMI, and how to improve equity in health financing between urban and rural residents are still in dispute. This paper attempts to describe the financing mechanism of NCMS and URBMI, decipher the differences in financing between NCMS and URBMI, analyze financing equity between urban and rural residents, and provide some comments on financing policies for integrating NCMS and URBMI into URRBMI.

## Methods

### Data sources

National documents on NCMS and URBMI were collected from the National Health and Family Planning Commission (NHFPC, including the former ministry of health, MOH) and the Ministry of Human Resources and Social Security (MOHRSS). Financing data on NCMS and URBMI were collected from the China health statistics yearbook (2009–2012), the China health and family planning statistics yearbook (2013–2016), the National Handbook of New Rural Cooperative Medical Scheme Information (2008–2015), the China human resources and social security statistics yearbook (2009–2016), and the China social security yearbook (2008–2015). The status of medical insurance enrollment and health services utilization for urban and rural residents were collected from the fourth and fifth national health household survey in China (2008, 2013). All secondary data could be available openly.

### Rationale of inequity measures in health financing

Equity is usually recognized by economists to be an important policy objective in the health care field [9–11]. The interest shown by economists in the equity issue seems to vary from one country to the next and over time within countries [9–15]. Williams compares and contrasts the libertarian and egalitarian positions. The egalitarian viewpoint suggests that a state sector should predominate, with health care being distributed according to “need” and financed according to “ability to pay”. The libertarian viewpoint, by contrast, points towards a mainly private health care sector, with health care being rationed primarily according to willingness to pay [10–12]. As China is a socialism country and holds the ideology of egalitarianism during the establishment of basic medical insurance systems, therefore, financing according to “ability to pay” was selected to measure equity in health financing [9–12] in this paper.

Financing health care according to ability to pay can be interpreted as vertical equity and horizontal equity, with the latter referring the same contribution by persons or families of the same ability to pay [10, 16]. As urban and rural residents in China shared similar characteristics of working in informal sector or unemployed, the rationale of horizontal equity between these two groups is that they are assumed to finance with the same share of their income. In this study, individual contributions to NCSM and URBMI as a percentage of per capita disposable income was selected to analyze inequity in health financing between rural and urban residents. According to statistics year book, the indicators to measure personal income is disposable income per capita for urban population and net income for rural residents. Expenditures on health care by urban households and rural households per capita were also collected to describe disparities in health financing between rural and urban residents.

## Results

### Coverage of NCMS and URBMI

Rural residents were mainly covered by NCMS. However, some rural residents who moved to urban areas were likely covered by URBMI at the same time. URBMI usually covered children in urban areas, employees in informal sectors, seniors in urban areas who weren’t covered by the pension system and immigrants from rural areas. NCMS covered more than 800 million rural residents from 2008 to 2013; however, this number decreased gradually due to the rapid urbanization of China. In 2015, 670 million rural residents were covered by NCMS (Table 1). URBMI covered increasing amounts of enrollees as urbanization increased. In 2008, the number of enrollees of URBMI totaled 118.26 million, and this total increased to 376.89 million in 2015 (Table 1).

**Table 1:** Number of the enrollees of NCMS and URBMI in China from 2008 to 2015

Year	URBMI (million)	NCMS (million)
2008	118.26	815.18
2009	182.1	833
2010	194.72	836
2011	221.16	832
2012	271.56	805
2013	296.29	802
2014	314.49	736
2015	376.89	670

Source: National Handbook of New Rural Cooperative Medical Scheme Information (2008–2015), China human resources and social security yearbook (2009–2016), China social security yearbook (2008–2015)

### Pooling fund and financing mechanism

The pooling fund per capita for URBMI was 140 CNY (USD20.44)^1^ in 2008 and decreased to 130CNY (USD 18.98) in 2009. Then, it increased annually and reached 515CNY (USD 79.31) in 2015. The percentage of URBMI’s pooling fund that came from individual contributions decreased from 45% in 2008 to 20.78% in 2014; then, it increased to 21.75% in 2015 (Table 2). Individual contributions to the pooling fund deviated from the policy requirement that families act as the key contributors to the URBMI fund.

**Table 2:** Financing structure of China’s URBMI and NCMS from 2008 to 2015

Year	URBMI			NCMS			
	Financing per capita (Ұ)	Individual contribution (%)	Contribution of the public finance (%)	Financing per capita (Ұ)	Individual contribution (%)	Contribution of the public finance (%)	Others (%)
2008	140	45.00	55.00	96.25	15.38	83.57	1.05
2009	130	39.23	60.77	113.37	20.56	78.53	0.91
2010	164	32.93	67.07	156.50	18.64	80.53	0.83
2011	246	25.20	74.80	246.21	14.73	84.38	0.89
2012	284	21.83	78.17	308.66	17.94	81.05	1.01
2013	360	21.67	78.33	370.63	18.09	80.70	1.21
2014	409	20.78	79.22	411.04	17.69	81.09	1.22
2015	515	21.75	78.25	490	19.25	79.83	0.92

Source: Adapted from National Handbook of New Rural Cooperative Medical Scheme Information (2008–2015), China human resources and social security yearbook (2009–2016) and China social security yearbook (2008–2015)

The pooling fund per capita for NCMS was 96.25 CNY (USD 14.04) in 2008 and gradually increased to 490CNY (USD75.46) in 2015. The percentage of NCMS’s pooling fund coming from individual contributions was 15.38% in 2008 and 19.25% in 2015. The lowest percentage was 14.73% in 2011, and the highest percentage was 20.56% in 2009 (Table 2). Public finance was a key financing source for the NCMS fund.

Government at different levels played various roles in financing the URBMI and NCMS funds. The central government was responsible for financing both URBMI and NCMS. The contribution of central government has been increasing, achieving 32.28% for URBMI and 57.22% for NCMS. Government at local levels, including provincial, prefectural and county government, provided subsides for these two insurance systems jointly. In URBMI, local government took more responsibility in financing than the central government. The role of the central government in financing NCMS became increasingly important, and its contributions to the NCMS fund has outweighed those of local governments since 2013 (Table 3). The central government tried to reduce the disparity between urban and rural areas by contributing more to the NCMS fund.

**Table 3:** Share of central government and local government’s financing as public finance contribution to URBMI and NCMS from 2008 to 2015

Year	URBMI (%)		NCMS (%)	
	Central government	Local governments	Central government	Local governments
2008	28.11	71.89	37.67	62.33
2009	28.95	71.05	36.36	63.64
2010	28.84	71.16	37.87	62.13
2011	33.49	66.51	44.68	55.32
2012	36.79	63.21	48.34	51.66
2013	35.27	64.73	51.39	48.61
2014	31.18	68.82	56.14	43.86
2015	32.28	67.72	57.22	42.78

Source: Adapted from National Handbook of New Rural Cooperative Medical Scheme Information (2008–2015) and China social security yearbook (2008–2015)

### Inequity in health financing

**Fig. 1:**
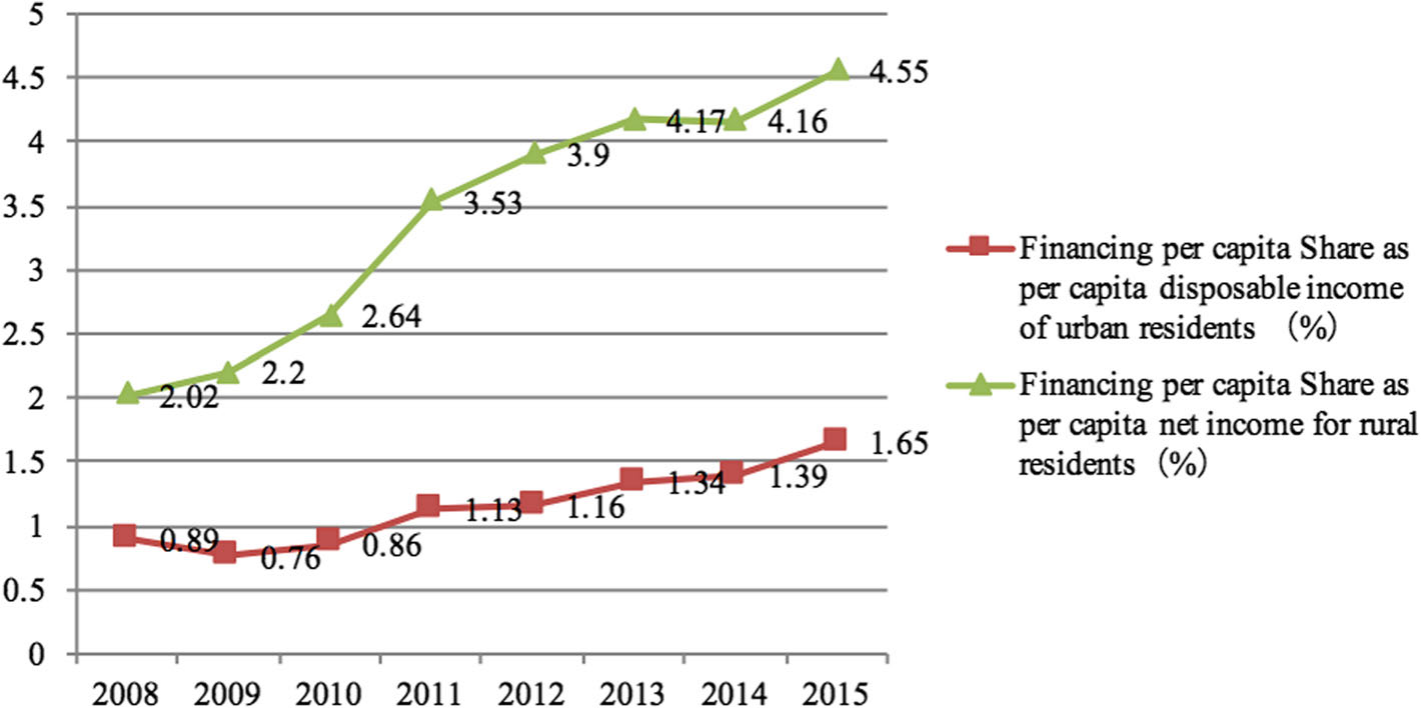
Share of financing per capita on NCMS and URBMI as disposable income of urban residents and net income for rural residents between 2008 and 2015

**Fig. 2:**
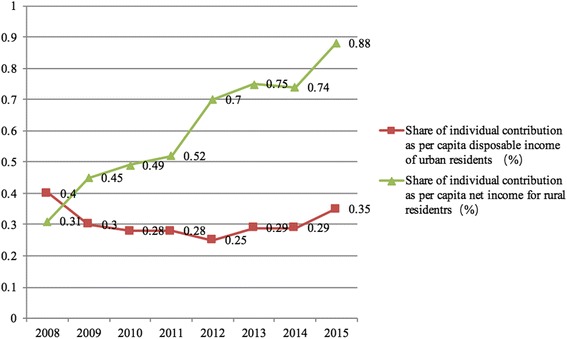
Share of individual contribution per capita to NCMS and URBMI as disposable income of urban residents and net income for rural residents between 2008 and 2015

The volume of financing per capita for URBMI and NCMS was very similar between 2008 and 2015, but they had different impacts on urban and rural residents in terms of disposable income and net income. The share of financing per capita as per capita disposable income of urban residents was kept at less than 2%, whereas it was higher than 2% for NCMS in 2008 and reached 4.55% in 2015 (Fig. 1). Urban residents’ individual contributions per capita to the URBMI fund were no more than 0.4% between 2008 and 2015, whereas rural residents’ individual contributions per capita to the NCMS fund were higher than that value for URBMI during the same period. The contributions per capita were 0.31% in 2008 and gradually increased to 0.88% in 2015 (Fig. 2). The gap between individual contributions to the URBMI and NCMS funds did not converge as expected, but rather diverged, as rural residents contributed more to the fund in terms of per capita disposable income or net income (Figs. 1 and 2).

**Fig. 3:**
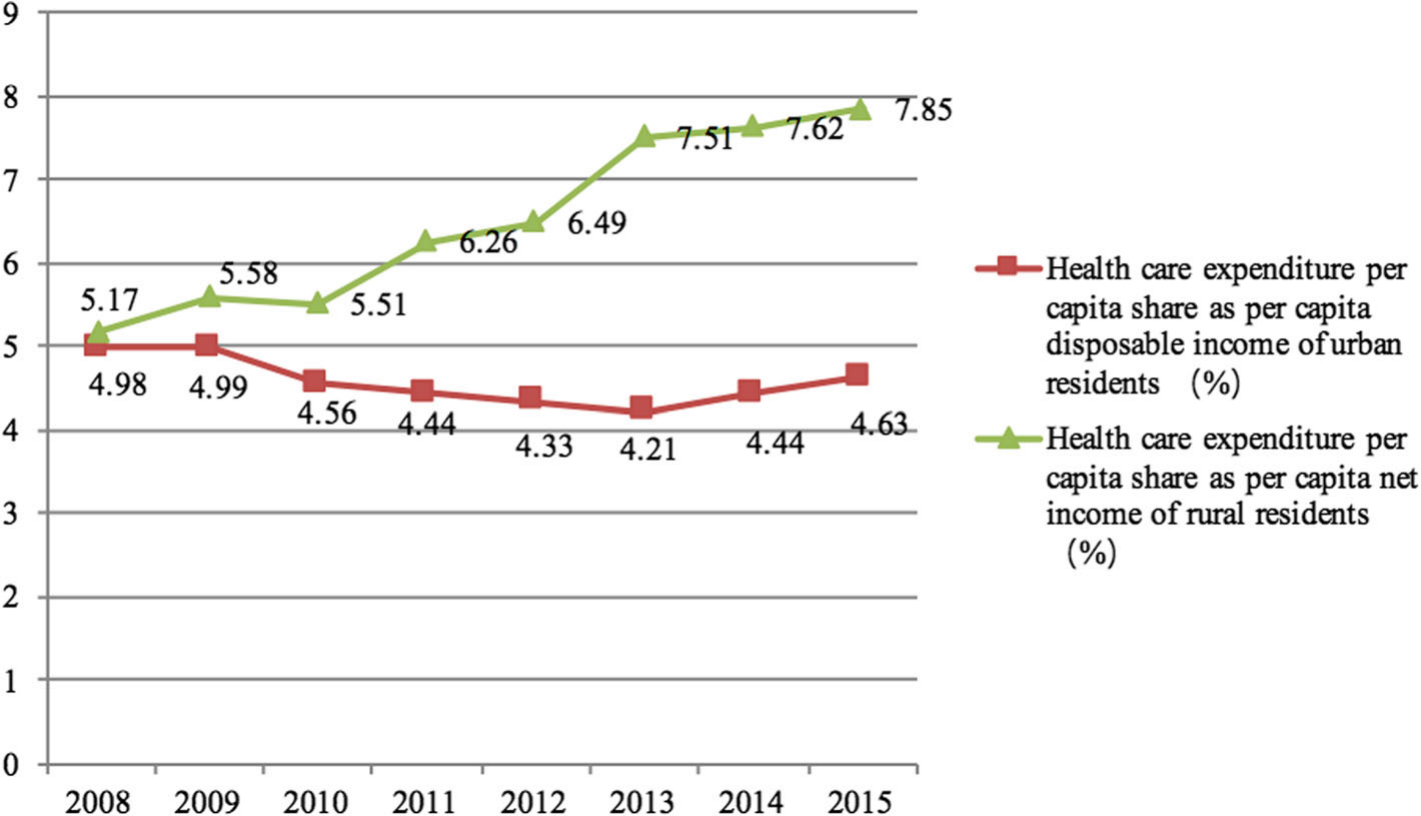
Share of health care expenditure per capita as disposable income of urban residents and net income for rural residents between 2008 and 2015

Health care expenditures and their share of household consumption expenditures for urban and rural residents were also compared to decipher the disparity between urban and rural residents. As Table 4 shows, health care expenditures per capita for urban and rural residents increased consistently from 2008 to 2015, and health care expenditures for urban residents were higher than those for rural residents. In 2015, they were 1443.4 CNY (USD 222.28) and 846.0CNY (USD 130.28), respectively. There is a different trend in the relationship between the shares of health care expenditures per capita as per capita disposable income for urban residents and as per capita net income for rural residents between 2008 and 2015. They fluctuate for urban residents, while they consistently rise for rural residents during this period. The share of health care expenditures per capita was 4.89% of the per capita disposable income of urban residents in 2008; then it decreased from 4.99% in 2009 to 4.21% in 2013; and increased to 4.63% in 2015. Meanwhile the share of health care expenditures per capita gradually increased from 5.17% in 2008 to 7.85% in 2015 as per capita net income for rural residents (Fig. 3).

**Table 4:** Comparison of China’s health care expenditure per capita for urban and rural residents between 2008 and 2015

Year	Health care expenditure per capita for urban residents (Ұ)	Health care expenditure per capita share as per capita disposable income of urban residents (%)	Health care expenditure per capita for rural residents (Ұ)	Health care expenditure per capita share as per capita net income of rural residents (%)
2008	786.20	4.98	246.0	5.17
2009	856.4	4.99	287.5	5.58
2010	871.8	4.56	326.0	5.51
2011	969.0	4.44	436.8	6.26
2012	1063.7	4.33	513.8	6.49
2013	1136.1	4.21	668.2	7.51
2014	1305.6	4.44	753.9	7.62
2015	1443.4	4.63	846.0	7.85

Sources: China statistics yearbook (2009–2016)

## Discussion

### The scale of financing in URRBMI

China succeeded in establishing NCMS and URBMI and covered more than 95% of informal employees and the unemployed by improving public investment, which won extensive attention and international recognition [17, 18]. There have been impressive advances in establishing medical insurance systems in China during the past decade, which has laid the foundation for China to be one of the countries to approach to universal health coverage [19–24]. However, the benefit packages of URBMI and NCMS could not meet the increasing demands for medical services by the insured, and many residents lacked access to health care due to economic reasons. According to the fifth national health household survey in China, in 2013, 9.1% of those insured by URBMI and 8.2% of the enrollees of NCMS were not admitted to hospitals in a timely fashion because of economic reasons [25]. One of the main reasons that urban and rural residents lacked access to health care was that the scale of financing was low for URBMI and NCMS. The scale of financing for NCMS was less than 5% of the per capita net income for rural residents, and it was lower than 2% of the per capita disposable income for urban residents. Compared to the fund for urban employees’ basic medical insurance, which collected at least 8% of the payroll and reached 3144CNY (USD 484.18) per capita in 2015 [26], the scale of financing for both URBMI and NCMS was insufficient, especially for URBMI.

### Disparity of individual contribution

URBMI and NCMS were integrated into URRBMI under the context of dualistic structures in urban and rural areas and the imbalance of health care resource allocation between urban and rural areas. And many provinces such as Guangdong, Shandong, Ningxia, Chongqing, Qinghai and Zhejiang provinces stick to current financing mechanism of NCMS and URBMI when URRBMI was integrated [27]. More attention should be paid to equity in health care financing, especially to reverse subsidies indicating that the rich (urban residents) benefit more from URRBMI than the poor (rural residents) [27, 28]. Based on the principle of “ability to pay”, rural residents contributed more to URRBMI than urban residents, and rural residents spent more than urban residents on health care in terms of per capita disposable income and net income between 2008 and 2015 in China. Reverse subsidies existed to some extent during the last 8 years and will continue to exist if the financing mechanism for URRBMI is not changed.

Individual contributions to the URBMI and NCMS funds were small in terms of incomes. Contributions were less than 1 % of the disposable income of urban residents and the net income of rural residents. In 2016, China’s new health guidelines proposed that health should be of the people and by the people [29]. It is helpful to implement new health guidelines through increasing individual contributions to the URRBMI fund and keep its development sustainable.

### The role of central and local governments in financing URRBMI

Encouragement of a higher budgetary allocation to health care remains a cornerstone of global health practice [30]. Health, an important sector of national welfare, will be the top priority in China in the near future [29]. However, to date, there are no institutional arrangements for the fiscal responsibility of contributions to the URRBMI fund in China. Contributions from public finance to the URRBMI fund depends on fiscal space of different levels of government, and uncertainty will continue to exist for the pooling fund of URRBMI under current circumstances. China is also in the process of reforming its fiscal governance system and is committed to unifying routine power and fiscal power properly [31]. However, how to adjust the roles of the central government and local governments regarding financing URRBMI has yet to be determined.

### Equity in health financing between urban and rural residents

URRBMI, as a component of social security and a stabilizer of economic and social development [32], aims to protect urban and rural residents from poverty due to illness, reduce disparities and improve the equity of health care financing between urban and rural areas. However, judging from the operations of URBMI and NCMS in the past decade, they did not reduce the disparity between urban and rural areas as expected but instead exacerbated the divergence of dualistic structures in urban and rural areas. The gap between urban and rural residents’ health care expenditures per capita as a share of per capita disposable income became divergent increasingly. Underlying reasons for this gap may be the higher access to health care for urban residents and relatively smaller benefit package for rural residents. This implied that financing policy for URRBMI should be adjusted so as to reduce the disparity between urban and rural residents.

### Characteristics of URRBMI

URRBMI was seen as a type of social medical insurance system in China, but it was against the main characteristic of a social medical insurance system. Compulsory enrollment is a key characteristic of social medical insurance systems around the world [33], but voluntary enrollment was introduced as a principle of URRBMI since the establishment of NCMS and URBMI [4, 6]. Individuals and employers or public finance co-sharing the premiums is another characteristic of social medical insurance systems [33], but the role of public finance has become increasingly important in the financing structure since the establishment of NCMS and URBMI in China. Based on the financing structure, URRBMI is approaching the levels of the National Health Service (NHS), which features tax-based health care financing [34].

### Limitations

With the limited availability of secondary data, inequity on health care financing between urban and rural residents was only measured at population level, not at personal level, comparing between urban and rural residents covered by URRBMI, which could reflect disparities between urban and rural residents to some extent. As China is a society under the context of dualistic structures and big difference still existed between urban and rural areas, and the difference of per capita disposable income of urban resident and rural residents were expanding 31,195 CNY (USD 4804.03) for urban and 11,422 CNY (USD 1758.99) for rural residents in 2015 [35], the results of inequity at population level may be deteriorated at personal level. More attention should be paid to inequity in health care financing among residents of different income stratifications. As China prepares to integrate NCMS and URBMI into URRBMI, nearly no research has focused on equity in health care financing between urban and rural residents; therefore, this paper did not compare the findings to those of other researchers.

## Conclusions

NCMS and URBMI played an important role in the process of approaching universal health coverage. China is committed to transforming from shallow and broad coverage to deep coverage of basic medical insurance systems. Integration of URRBMI is an important component to achieving this goal. However, more steps need to be taken to determine the financing mechanism of URRBMI.

The financing scale of URRBMI should be increased so that it can better adjust to the rapidly aging population in China, adjust to epidemiological transitions, meet the increasing demands for health care by urban and rural residents and protect them from poverty due to illness. The scale of financing determines the benefit packages and cost sharing. The bigger the financing scale of URRBMI becomes, the broader the benefit packages become, and the more cost sharing is for the insured.

Financing per capita for URBMI and NCMS was very equal in the last decade, while inequity was veiled by equality. Inequity in health care financing should be improved between urban and rural residents under the circumstance of balancing between urban and rural development. Inequity in health care financing between urban and rural residents did not become convergent under the current financing mechanism in China. There is still much room for improvement in inequity in health care financing between urban and rural residents in China. The financing mechanism of URRBMI should be adjusted based on the principle of “ability to pay” so that it reduces the disparity between urban and rural residents in China.

Compulsory enrollment should be instituted so that URRBMI better transforms to the social medical insurance system. Under the new normal of China’s economy, the growth rate of public finance will decreased as expected, and public finance cannot contribute to the fund of URRBMI in line with the rapidly increasing demands for health care by urban and rural residents simultaneously. Individual contributions to the URRBMI fund should be increased so that URRBMI can develop sustainably.

The role of the central government and local governments in financing URRBMI was not clearly identified. Public contributions to the URRBMI fund should be clearly identified with the principle of unifying authority over the administrative and financial affairs so that a sustainable financing mechanism can be established.

## Footnotes

1 The exchange rate of Chinese yuan to USD was 0.146 on 31 Dec. 2008, and 0.146 on 31 Dec. 2009, 0.154 on 31 Dec. 2015 based on OANDA.
